# Mechanisms and therapeutic design of stimuli-responsive vesicular nanocarriers for melanoma

**DOI:** 10.1016/j.ijpx.2026.100638

**Published:** 2026-08-12

**Authors:** Rudresh Adarkar, Akanksha Dessai, Manoj Madanahalli Ramesh, Richard Lobo, Vamshi Krishna Tippavajhala, Chandrashekar Kodangala Subraya

**Affiliations:** a Department of Pharmacognosy, Manipal College of Pharmaceutical Sciences, Manipal Academy of Higher Education, Manipal, India; b Department of Pharmaceutics, Manipal College of Pharmaceutical Sciences, Manipal Academy of Higher Education, Manipal, India

**Keywords:** Melanoma, Transdermal drug delivery, Stimuli-responsive nanocarriers, Tumor microenvironment, Lipid vesicles, Reactive oxygen species, Matrix metalloproteinases

## Abstract

Cutaneous melanoma is an extremely aggressive form of cancer and a major cause of death worldwide. Localized delivery by transdermal application is an alternative to systemic chemotherapy, which has a low therapeutic index and off-target toxicity. The clinical translation of topical oncology is hindered by a formidable dual barrier: the highly ordered and rigid lipid-protein barrier of the stratum corneum (SC) and the fibrotic, restrictive tumor microenvironment (TME) of the melanoma. In this review, the paradigm shift from passive delivery vehicles to stimuli-responsive vesicular nanocarriers, which are systematically designed to overcome these physical and biochemical barriers, is critically explored. We critically analyse the bioengineering of stimuli-responsive lipidic systems, such as nanovesicular systems, that preserve the deformability of the SC whilst leveraging specific pathophysiological features of the melanoma TME. Moreover, in order to close the loop between elegant concepts and clinical feasibility, we specifically discuss the major translational hurdles that currently hinder this field, such as manufacturing scalability issues, regulatory hurdles, and the lack of adequate standard murine models. Key structural modifications are discussed, including the use of extracellular acidosis via charge reversal and acid-cleavable linkages, oxidative stress via ROS-responsive thioether and thioketal phase transitions, and enzyme overexpression via matrix metalloproteinase (MMP)-triggered de-PEGylation. These intelligent nanocarriers offer a very promising approach to achieving maximum intracellular accumulation by enabling on-demand payload release at a spatiotemporally controlled location within the malignant stroma. Such responsive systems are shown to routinely produce significant enhancements in deep transdermal permeation and even greater enhancements in local cytotoxicity than passive liposomes. Finally, how this strategy is able to overcome the restrictions of the heterogeneous enhanced permeability and retention (EPR) effect and substantially enhance the therapeutic index of targeted melanoma therapy.

## Highlights

• Stimuli-responsive vesicles overcome the dual SC and melanoma TME barrier.
• pH-responsive charge reversal enables tumor-specific on-demand drug release.
• ROS-sensitive thioether and thioketal linkers drive selective vesicle disassembly.
• MMP-triggered de-PEGylation unmasks targeting ligands within malignant stroma.
• Translational bottlenecks in scalability and preclinical models are critically addressed.

## Graphical abstract

**Figure:**
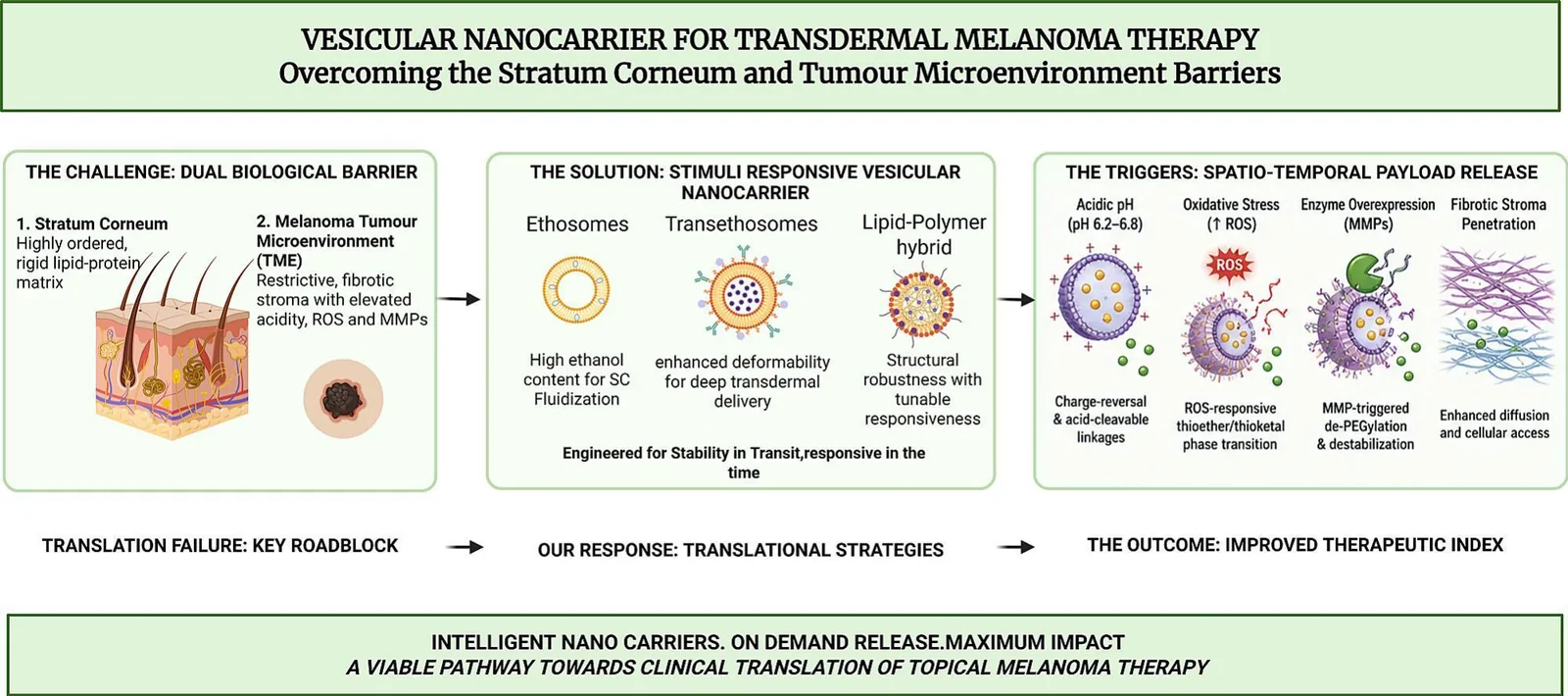

## 1 Introduction

**Fig. 1:**
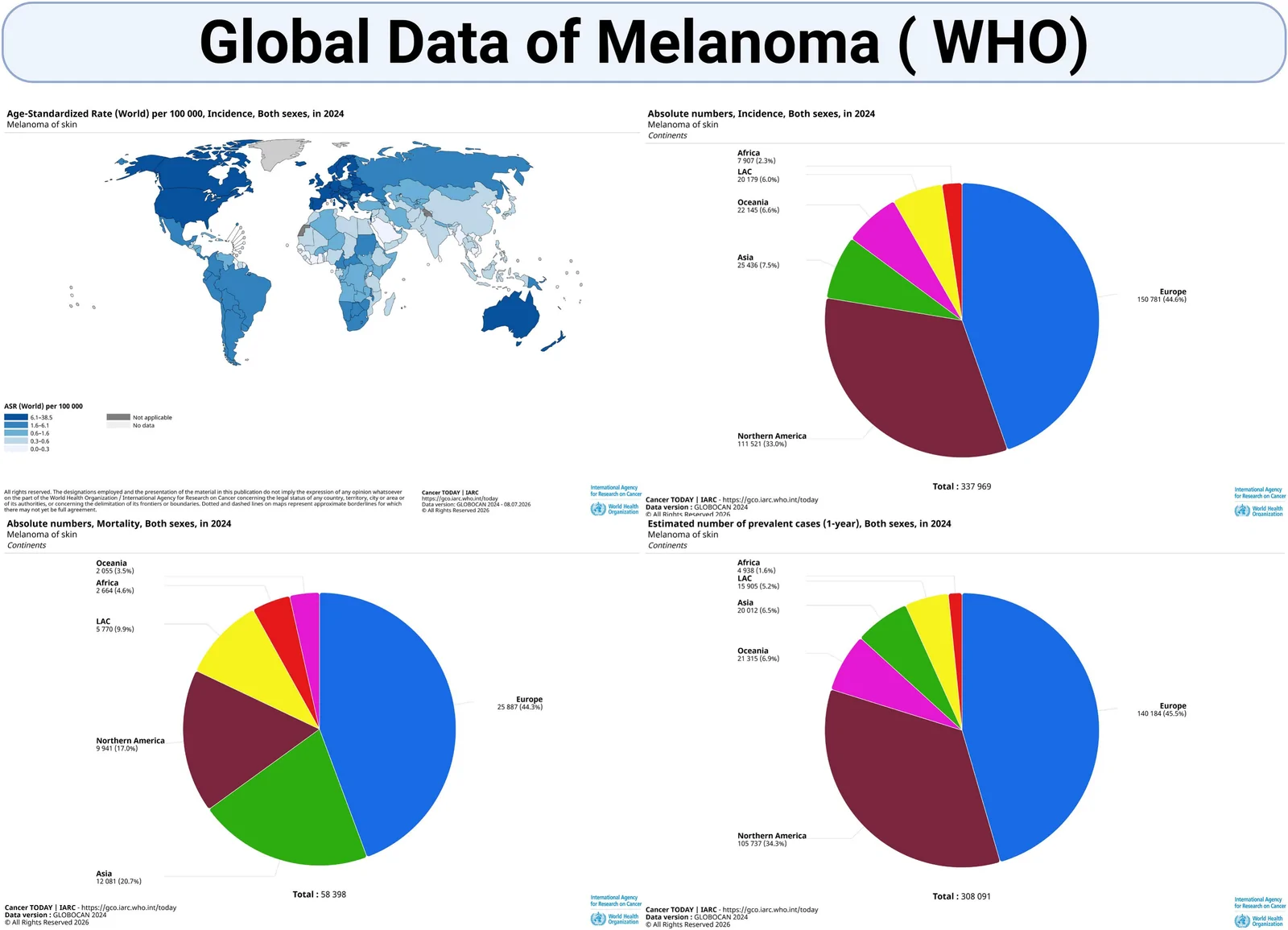
Global prevalence data of melanoma skin cancers. The figure presents WHO GLOBOCAN 2024 melanoma epidemiology data. The top-left panel depicts a global choropleth map of the age-standardised incidence rate (World, per 100,000 population) for melanoma of skin. The top-right, bottom-left, and bottom-right panels present pie charts illustrating the continental distribution of absolute incident cases (total: 337,969), absolute mortality (total: 58,398 deaths), and estimated 1-year prevalent cases (total: 308,091), respectively. Collectively, the data demonstrate a higher overall incidence burden in Europe and other high-income regions, contrasted with a disproportionately higher mortality burden in Asia, which accounts for 20.7% of global melanoma deaths despite representing only 7.5% of incident cases, thereby highlighting regional disparities in early detection, disease awareness, and access to medical treatment.

Skin cancer or melanoma poses a significant and increasing burden on the global oncology infrastructure. Among cutaneous malignancies, melanoma is responsible for most of the skin cancer-associated mortality due to its extremely aggressive progression and rapid metastatic spread (Dee et al., 2025). The need for advanced, localized interventions is underscored by the growing global epidemiological burden of the disease. According to the latest GLOBOCAN 2022 estimates, 331,722 new cases of malignant melanoma were diagnosed globally, resulting in approximately 58,667 deaths (Bray et al., 2024). This incidence is not uniformly distributed, as the regions such as Australia, North America, and Northern Europe report disproportionately high rates, driven by a combination of genetic susceptibility and cumulative environmental exposure. Furthermore, epidemiological forecasting suggests a continued upward trajectory in global incidence over the next two decades. While early-stage, localized melanoma boasts a high five-year survival rate following surgical excision, the prognosis precipitously declines once the malignancy affects the deeper dermal layers and disseminates. The distinct lethality of advanced malignancy, coupled with its rising global prevalence, highlights a critical urgency to shift the therapeutic paradigm toward highly targeted interventions capable of navigating the complex biology of the melanoma lesion. (Bray et al., 2024; Chen and Liu, 2025; Urban et al., 2021; Wang et al., 2025). Global epidemiological data for cutaneous melanoma are summarised in Fig. 1. Unless otherwise specified, all epidemiological figures and statistics cited throughout this review refer exclusively to cutaneous melanoma and not to non-melanoma skin cancers.

Currently, systemic chemotherapy for cutaneous malignancies is severely slowed down by a critically low therapeutic index. Due to the widespread systemic volume of distribution and rapid hepatic clearance, only a small fraction of the intravenously administered dose reaches the cutaneous tumor cells. Consequently, systemic protocols frequently fail to achieve the minimum effective concentration required within the tumor microenvironment without first inducing dose-limiting, off-target systemic toxicities (Grantab and Tannock, 2012; Jin et al., 2025; Shen et al., 2024). This pharmacokinetic inefficiency is contributed by systemic drug dilution and the skin's vascular architecture, which restricts the extravasation (accidental leakage) of circulating chemotherapeutics into the dermal and epidermal tumor microenvironment (Eggermont and Eggermont, 2024). Consequently, there is an urgency to change the therapeutic paradigm to a localized, non-systemic treatment that could focus cytotoxic or immunomodulatory therapies at the site of neoplastic disease and avoid healthy systemic physiology (Sun et al., 2023).

Topical and transdermal drug delivery offer a highly advantageous physiological pathway for localized therapy of skin cancers(Yourdkhani et al., 2025). The topical route bypasses hepatic first-pass metabolism, reduces systemic exposure, and enables sustained, high-concentration dosing within the tumor architecture(Pham and Tsunoyama, 2024). Despite these pharmacological advantages, formidable biophysical barriers have severely limited the clinical translation of topical oncology. The primary limitation is the stratum corneum (SC), an evolutionarily conserved, highly ordered lipid-protein matrix that acts as a potent barrier, subsequently restricting the permeation of high-molecular-weight and hydrophilic chemotherapeutics (Gupta et al., 2022).

Furthermore, when conventional chemical enhancers/standard passive nanocarriers for penetration (e.g., liposomes) are used, the systems often doesn't normally distinguish between normal dermal tissue and malignant cells, further resulting in localized toxicity and suboptimal intracellular accumulation in the tumor (Peralta et al., 2018). To overcome this challenge, that is, avoid the strict SC and yet release the therapeutic agent specifically within the malignant tissue, the design of drug delivery systems should transform itself to engineered, intelligent nanocarriers as opposed to passive vehicles(Yourdkhani et al., 2025). The present review critically analyses recent developments in stimuli-responsive vesicular delivery systems and how strategically engineered lipid vesicles can leverage pathophysiological triggers in the melanoma microenvironment to produce local therapeutic effects never before observed(Secci et al., 2026).

## 2 The dual biological barrier: the stratum corneum and the melanoma tumor microenvironment

The localized delivery of chemotherapeutics for cutaneous malignancies presents a unique, dual biophysical challenge. Nanocarriers must first breach the skin's outer layer before navigating the complex, dynamic, and physically restrictive stroma of the TME (Murphrey et al., 2022). Understanding and quantifying these dual barriers is a foundational necessity for designing advanced, stimuli-responsive vesicular systems (Yu et al., 2025).

### 2.1 The primary barrier: the stratum corneum

The SC is the uppermost layer of the epidermis and is the main rate-limiting barrier in transdermal oncology (Benson et al., 2019). The SC, which is anatomically modeled as a brick-and-mortar structure, is composed of terminally differentiated and nucleated corneocytes surrounded by a highly organized intercellular matrix of lipophilic compounds. This matrix is predominantly made up of ceramides, cholesterol, and free fatty acids in an approximately equimolar (1:1:1) ratio (Murphrey et al., 2022). This stiff structure under physiological conditions limits the permeation of molecules with a molecular weight over 500 Da or molecules that are highly hydrophilic (Abdul Aziz et al., 2025; Bos and Meinardi, 2000; Ramadon et al., 2022). Therefore, most regular chemotherapeutic agents (e.g., doxorubicin, paclitaxel, 5-fluorouracil) exhibit insignificant passive permeation(Aminipour et al., 2020; Liu et al., 2015). Though it is possible to break this barrier with physical permeation enhancers (microneedles or iontophoresis), they usually result in local erythema and do not provide sustained, subcellular targeting of deep-seated melanomas(Jiang et al., 2025; Nemakhavhani et al., 2025). Therefore, the optimal nanocarrier should have high deformability and fluidization to squeeze through the intact intercellular lipid domains of the SC without undergoing abrasive physical breakdown.

### 2.2 The secondary barrier: the restrictive melanoma stroma

**Fig. 2:**
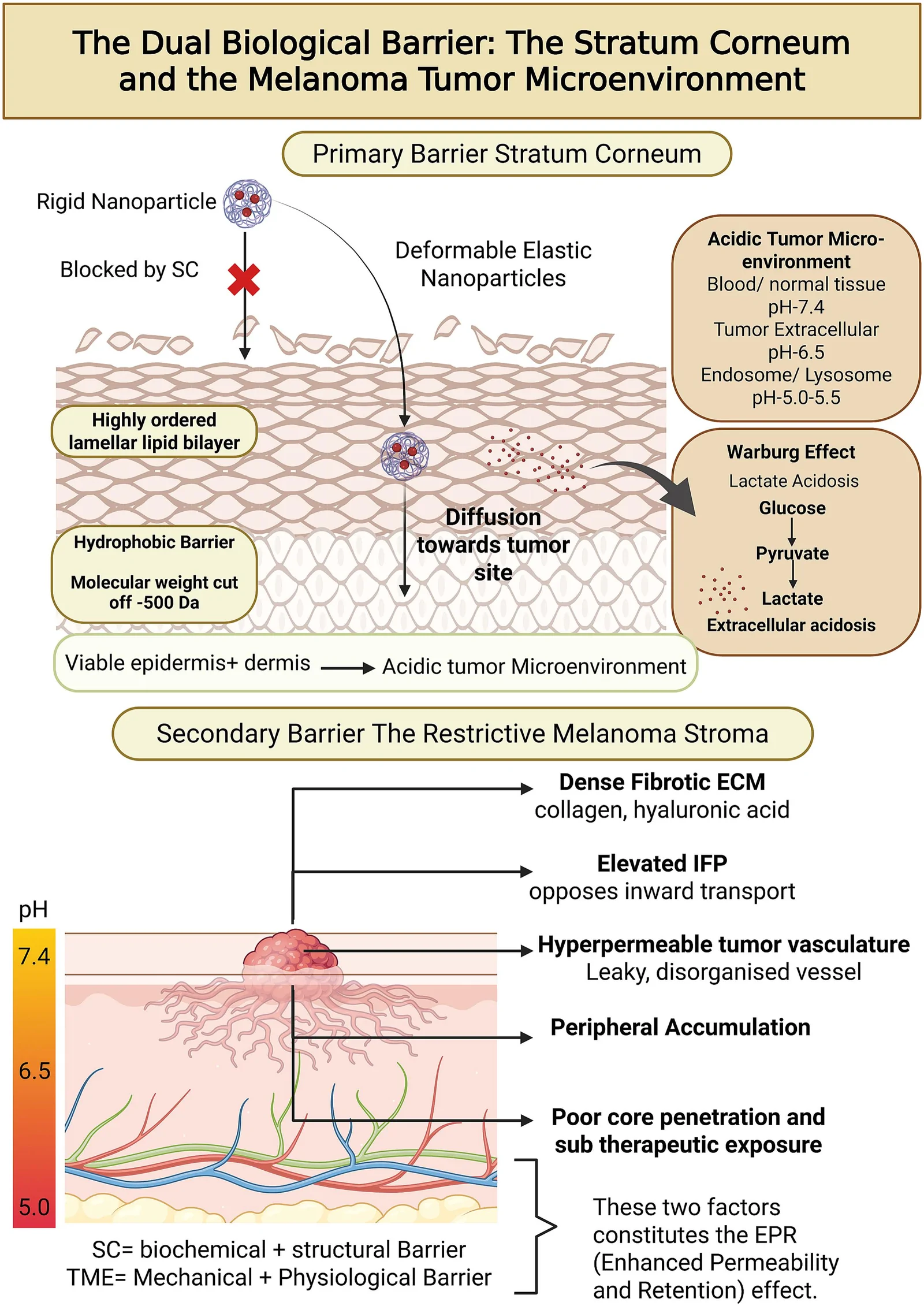
The dual biological barrier in topical melanoma therapy. The schematic shows the two main challenges associated with transdermal nanotherapeutics. The main obstacle is the highly ordered lipidic SC that allows the passage of highly deformable elastic vesicles but not rigid nanoparticles. As they pass through the SC, they will come across the second barrier, the restrictive melanoma TME. The TME is characterized by a dense fibrotic ECM and high IFP, which is opposing the inward transport and makes the EPR effect more difficult. At the same time, a distinct pathophysiological pH gradient is triggered by the Warburg effect: the extracellular pH drops from the physiological level (7.4) to an extracellular acidosis (∼6.5) and even to severe endosomal/lysosomal acidosis (5.0) which is a primary trigger for engineered stimuli-responsive systems.

When nanocarriers penetrate the epidermis successfully, they encounter the second barrier: the dense, highly pressurized melanoma TME. In contrast to normal dermal tissue, solid tumors are characterized by disorganized, hyperpermeable angiogenesis and deficiency of functional lymphatic drainage- a phenomenon more traditionally known as the EPR effect (Jasim et al., 2017). However, a dense, fibrotic extracellular matrix (ECM) rich in collagen and hyaluronic acid strongly opposes the EPR effect in melanoma, significantly elevating the interstitial fluid pressure (IFP) within the tumor core (Huang et al., 2024; Romano et al., 2021). This elevated IFP successfully halts the passive inward diffusion of both soft vesicular systems, such as conventional liposomes, and rigid carriers, such as solid polymeric or metallic nanoparticles. Consequently, these carriers accumulate on the periphery of the tumor instead of infiltrating the necrotic core, resulting in sub-lethal dosing and acquired chemoresistance (Jiang et al., 2024). Both these barriers are explained using a schematic diagram in Fig. 2.

### 2.3 Limitations of passive diffusion and the EPR effect in melanoma

Drug delivery vehicles need to have appropriate properties to effectively traverse both the dual-barrier properties of the SC and the melanoma TME (Gupta et al., 2022; Pandey et al., 2021). They should be rigid and flexible enough to permeate the intact epidermal lipid matrix without early drug release, but very much sensitive and degradable upon entering the neoplastic stroma to achieve rapid release of intracellular payloads (Alam et al., 2023; Boakye et al., 2016; Motawea et al., 2024). Traditional passive nanocarriers, such as standard liposomes, solid lipid nanoparticles (SLNs), and simple polymeric micelles, often fail to meet these differing demands.

The EPR effect has been extensively studied in the context of passive nanocarriers for oncology. Nonetheless, clinical consensus in the recent past has noted that the EPR effect is very heterogeneous and often blocked in melanoma by high interstitial fluid pressure in the tumor and high desmoplastic stroma density (Khan et al., 2026; Park et al., 2019; Wu, 2021). Passive nanocarriers can accumulate in the peritumoral space upon entry into the skin, subsequently leading to reduced cellular uptake, acquired drug resistance, and off-target toxicity in adjacent healthy dermal fibroblasts (BAZAK et al., 2014).

To overcome these fundamental limitations associated with passive diffusion and the heterogeneous EPR effect, pharmaceutical research has transitioned toward the rational design and development of smart/stimuli-responsive nanocarriers (Mi, 2020). These systems are no longer based upon simple concentration gradients but use dynamic cleavable chemical linkages or conformationally adaptable polymers incorporated into their structural matrix or surface coating. This architectural design ensures that the nanocarrier is in a locked and stealth state when passing through healthy skin layers, preventing the chemotherapeutic and immunomodulatory load from being cleared prematurely (Singh and Kumar, 2025; Thambi et al., 2016).

These smart nanocarriers are characterized by the ability to experience abrupt physicochemical changes, i.e., rapid swelling, surface charge reversal, membrane destabilization, or complete disassembly, only in response to the aberrant pathophysiological stimuli of the melanoma TME (Kenchegowda et al., 2021; Yeduvaka et al., 2026). Nanocarriers, which react to stimuli and are targeted at a specific tumor site, can be used to release drugs with high accuracy and on demand by exploiting the localized, spatiotemporal hallmarks of the tumor site (acidosis, oxidative stress, and enzyme overexpression) as biological keys, dramatically enhancing the therapeutic index of localized melanoma therapies (Zhao et al., 2021).

### 2.4 The TME as a therapeutic trigger: exploiting pathophysiological hallmarks

Early formulation approaches viewed the dense ECM merely as a potent physical inhibitor, thereby failing to leverage its unusual biochemistry for localized drug release. But recent advanced strategies actively exploit the aberrant pathophysiological hallmarks of the melanoma TME rather than merely trying to physically bypass them as highly specific biological triggers for on-demand payload delivery (Popovic and Tartare-Deckert, 2022). The rate of growth of melanoma cells exceeds the vascular supply, leading to clear metabolic and enzymatic phenotypes that sharply contrast with those of the surrounding healthy tissue (Fischer et al., 2017; Sobiepanek et al., 2021).

Extracellular Acidosis (The Warburg Effect) is such that Melanoma cells depend heavily on aerobic glycolysis, leading to excessive production and secretion of lactic acid into the extracellular space(Daverio et al., 2023a). While the physiological pH of healthy tissue is maintained at approximately 7.4, the extracellular pH (pHe) of the melanoma TME generally decreases to an acidic range of 6.2–6.8 (Boussadia et al., 2018; Chiheb et al., 2025). Furthermore, upon cellular internalization, the pH drops precipitously to 4.5–5.5 within the intracellular endosomal and lysosomal compartments. This steep, highly localized pH gradient serves as the primary biochemical stimulus for the activation of pH-responsive vesicular systems(Böhme and Bosserhoff, 2016; Sutoo et al., 2019).

High levels of Reactive Oxygen Species (ROS)in melanoma cells are such that the low oxygen levels in the tumor center induce mitochondrial dysfunction that results in an enormous production of ROS, mostly hydrogen peroxide and hydroxyl radicals(Aggarwal et al., 2019; Huang et al., 2021). The intracellular ROS of melanoma may be 10–100-fold greater than that of normal melanocytes, which presents a highly specific chemical induction of ROS-scavenging or ROS-cleavable nanocarriers (Arslanbaeva and Santoro, 2020; Zhang et al., 2022b).

Melanoma cells and tumor-associated macrophages release large amounts of matrix-remodeling enzymes, promoting invasion and metastasis. There is a massive overexpression of matrix metalloproteinases (MMP-2 and MMP-9) and hyaluronidases in the cutaneous TME(Szczygielski et al., 2024). When peptide or polymer linkers are incorporated into the surface of the nanocarrier, which are specifically cleaved by these enzymes, it is possible to have an on-demand degradation and release of the vesicles only when they encounter the malignant stroma.

By mapping these pathophysiological stimuli, formulation scientists can design lipid bilayers for smart vesicular systems to be highly stable in the face of SC penetration, only to structurally collapse and release their payload upon exposure to the acidic, oxidative, or enzyme-rich environment of the melanoma TME.

## 3 Stimuli-responsive modification technologies for localized delivery

### 3.1 pH-responsive vesicular systems

The most widely studied stimuli-responsive approach in oncology is the exploitation of extracellular acidosis, which is mediated by the Warburg effect (Barba et al., 2024). The extracellular pH (pHe) is generally decreased to 6.2–6.8, and the intracellular endosomal and lysosomal compartments are even more acidic (pH 5.5–4.5) in the context of melanoma (Böhme and Bosserhoff, 2016; Peppicelli et al., 2024; Sutoo et al., 2019). To take advantage of this gradient, lipid nanocarriers are typically designed with one of two main structural features: either the incorporation of protonatable lipids/polymers or the use of acid-cleavable chemical linkages (Daverio et al., 2023b). This design rationale continues to anchor pH-responsive nanocarrier development across oncology broadly, as recently reviewed by Tapponi et al.(Tapponi et al., 2025).

#### 3.1.1 Protonatable systems and charge reversal

**Fig. 3:**
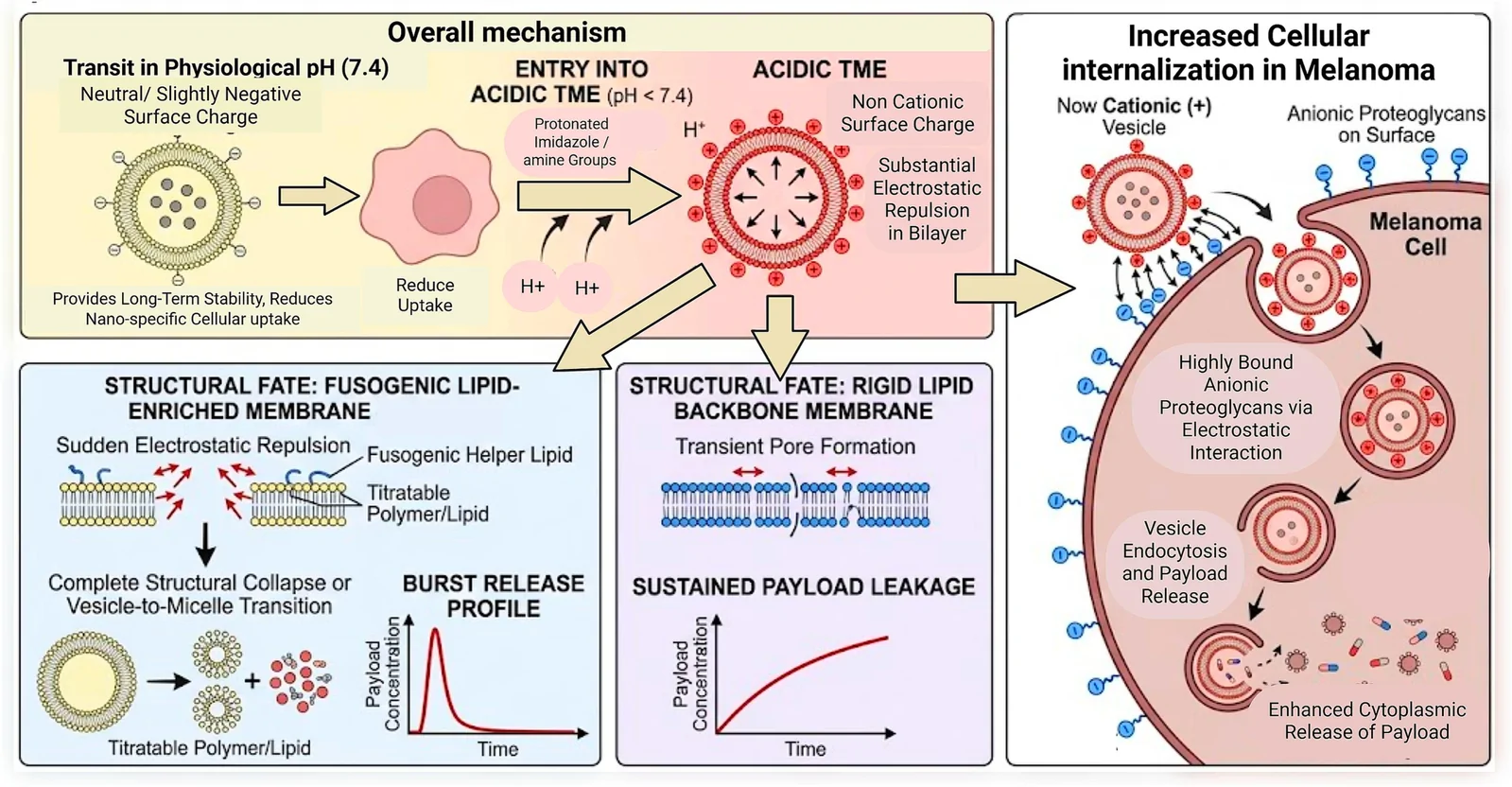
Mechanism of pH-Responsive Charge Reversal in Vesicular Nanocarriers. Illustration of the structural transformation of pH-responsive nanocarriers upon transition from physiological pH to the acidic TME. Vesicles maintain a neutral or slightly negative charge during transit to minimize nonspecific uptake. Upon exposure to the acidic TME, protonation of specific groups induces substantial electrostatic repulsion within the lipid bilayer. This results in either complete structural collapse for burst release (in fusogenic lipid-enriched membranes) or transient pore formation for sustained payload leakage (in rigid lipid backbones). The resulting cationic charge significantly enhances cellular internalization by malignant melanoma cells via electrostatic binding to anionic surface proteoglycans.

The vesicular membrane is typically formulated with specific titratable polymers such as poly(L-histidine) or glycol chitosan or advanced ionizable amino lipids, including 1,2-dioleoyl-3-dimethylammonium-propane (DODAP) and DLin-MC3-DMA, to form charge-reversible vesicles(Zhang et al., 2022a). These systems have a neutral or slightly negative surface charge at physiological pH (7.4), which provides long-term stability and reduces nonspecific cellular uptake during transit. However, upon exposure to the acidic TME, the imidazole rings or amine groups undergo rapid protonation. This sudden influx of positive charge induces substantial electrostatic repulsion within the lipid bilayer. The ultimate structural fate of the vesicle is highly codependent on its specific lipid composition. Formulations enriched with fusogenic helper lipids (such as DOPE) typically undergo a complete structural collapse or vesicle-to-micelle transition, which result in in a burst release profile, whereas vesicles with highly rigid lipid backbones may only undergo transient pore formation, facilitating a more sustained payload leakage (Cheng et al., 2023; Sabeel and Yang, 2025; Zhang et al., 2018). Moreover, this charge reversal significantly increases cellular internalization of the nanocarrier, since the now-cationic vesicles are highly bound to the anionic proteoglycans on the surface of melanoma cells (Zhuo et al., 2020). The mechanism is depicted in Fig. 3.

#### 3.1.2 Acid-cleavable linkages

Another, and much more specific, alteration is the conjugation of shielding polymers, most commonly polyethene glycol (PEG), to the vesicular surface via acid-sensitive bonds, such as hydrazone, orthoester, or imine bonds (Fang et al., 2017). PEGylation is essential to increase the deformability and stability of highly deformable vesicles during SC penetration, but the PEG dilemma states that the same stealth coating has an overwhelming effect on cellular uptake and endosomal escape after the carrier has been delivered to the tumor (Kong et al., 2019). Facilitated by the acid-labile hydrazone bond, the PEG corona is rapidly cleaved exclusively within the acidic stroma. This de-PEGylation reveals underlying targeting ligands or cationic lipids, which can be directly endocytosed by the malignant cells (Elsayed, 2024; Zhao et al., 2017).

#### 3.1.3 Resolving the transdermal bio-relevance gap

Although pH-responsive systems are highly effective for intravenous delivery, their use in transdermal oncology poses a major biophysical paradox that is often overlooked in the current literature on exploratory studies. The healthy human epidermis has a surface pH of 4.5–5.5, maintained by the acid mantle (Alsawaftah et al., 2022; Schafer et al., 2023). Thus, a typical pH-responsive nanocarrier designed to disassemble at a tumor extracellular pH of 6.5 would undergo premature structural degradation when first applied topically to the acidic mantle (Chu et al., 2022).

To overcome this bio-relevance gap, further formulation strategies should target intracellular pH stimuli rather than extracellular ones. Optimized transdermal nanocarriers should be designed to survive the slightly acidic passage through the SC and the near-neutral viable epidermis, intact until they are internalized by the melanoma cells, where the precipitous drop to lysosomal pH (<5.0) ultimately induces endosomal escape and cytotoxic release (Liu et al., 2023b; Qiu et al., 2023; Zhang et al., 2026).

Notably, extracellular-pH-triggered systems that have achieved in vivo efficacy have typically relied on either systemic/intratumoral administration or on physical stratum corneum bypass (e.g., microneedle-assisted delivery), circumventing rather than overcoming the topical bio-relevance gap; endosomal-pH-triggered systems, by contrast, are inherently compatible with topical application since their trigger is encountered only post-internalization. As a practical design rule, extracellular-pH-responsive systems should therefore be reserved for administration routes that bypass the acidic skin surface, systemic, intratumoral, or microneedle-assisted delivery, while passive topical formulations should be engineered to respond to intracellular endosomal or lysosomal pH rather than extracellular tumor pH.

### 3.2 ROS-responsive vesicular systems and how to exploit oxidative stress via thioether and thioketal linkers

In addition to the extracellular acidosis, the melanoma TME is also characterized by high oxidative stress. Mitochondrial dysfunction usually occurs due to rapid proliferation and metabolic dysregulation caused by hypoxia, leading to excessive formation of ROS, such as hydrogen peroxide (H_2_O_2_), superoxide (O_2_^−^), and hydroxyl radicals (OH) (Kaminski et al., 2022). The levels of intracellular ROS in melanoma cells are claimed to be 10–100 times greater than those of normal melanocytes (Liu-Smith et al., 2014; Zhang et al., 2022b), creating a strong endogenous stimulus to redox-responsive drug delivery systems.

Taking advantage of this biochemical gradient, ROS-responsive nanocarriers are designed by loading oxidatively sensitive functional groups into the vesicular core or by surface modification. Among the many chemistries studied, including disulfides, boronic esters, and proline-rich linkers, thioether and thioketal features have proven to be more structurally adaptable and more translationally relevant in vesicular oncology (Rinaldi et al., 2022; Tao and He, 2018; Zhang et al., 2024a).

Thioether and thioketal linkers react through different but complementary oxidation processes:

#### 3.2.1 Thioether-mediated phase transition

Thioether moieties, often incorporated in hydrophobic lipid domains or lipid-polymer hybrids (e.g., poly(propylene sulfide)), are oxidized by the presence of high H_2_O_2_ concentrations in steps to form sulfoxides and eventually sulfones (Liu et al., 2023a). This oxidative conversion significantly enhances the local polarity of the lipid domains, driving a rapid hydrophobic-to-hydrophilic transition. Thermodynamically, this localized hydration increases the effective cross-sectional area and hydration volume of the previously hydrophobic chains. This shift fundamentally disrupts the critical packing parameter (CPP) of the bilayer; as the oxidized lipids transition from a stable cylindrical geometry (CPP ≈ 1) required for lamellar vesicles to a normal cone shape (CPP < 0.5), the membrane experiences extremely high spontaneous positive curvature stress. This severe structural destabilization subsequently forces vesicle swelling, complete micellization, and the rapid, burst release of the encapsulated payload (Liu et al., 2023b; Saravanakumar et al., 2016).

The conversion proceeds via two consecutive oxidation steps mediated by hydrogen peroxide ($H_{2}O_{2}$), a major reactive oxygen species (ROS) present in the tumor microenvironment (Subraveti et al., 2024; Van Der Vlies et al., 2021).

Step 1: Oxidation of Thioether to Sulfoxide

$$R-S-R^{\prime }+H_{2}O_{2}\to R-S\left(O\right)-R^{\prime }+H_{2}O$$

Where:

• $R-S-R^{\prime }$= thioether moiety (hydrophobic)
• $R-S\left(O\right)-R^{\prime }$= sulfoxide intermediate

Step 2: Oxidation of Sulfoxide to Sulfone

$$R-S\left(O\right)-R^{\prime }+H_{2}O_{2}\to R-S{\left(O\right)}_{2}-R^{\prime }+H_{2}O$$

Where:

• $R-S{\left(O\right)}_{2}-R^{\prime }$= sulfone product (highly polar)

Overall Reaction

$$R-S-R^{\prime }+2H_{2}O_{2}\to R-S{\left(O\right)}_{2}-R^{\prime }+2H_{2}O$$

#### 3.2.2 Thioketal-cleavable linkers

Cleavage of bonds with thioketal: Conversely, thioketal linkers experience irreversible cleavage by ROS and are typically used to conjugate polyethylene glycol (PEG) coronas to vesicles (Rinaldi et al., 2022).

The degradation of thioketal linkers under oxidative conditions proceeds via ROS-mediated sulfur oxidation, followed by hydrolytic fragmentation of the carbon–sulfur framework (Pan et al., 2020; Parshad et al., 2025).

Step 1: Oxidation of Thioketal Sulfur Atoms to Sulfoxides

$$\left(R){}_{2}C{\left(SR^{\prime }\right)}_{2}+2H_{2}O_{2}\to {\left(R\right)}_{2}C{\left[S\left(O\right)R^{\prime }\right]}_{2}+2H_{2}O\right)$$

Where:

• ${\left(R){}_{2}C(SR^{\prime }\right)}_{2}$= thioketal linker
• ${\left(R\right)}_{2}C{\left[S\left(O\right)R^{\prime }\right]}_{2}$= sulfoxide intermediate

Step 2: Hydrolytic Cleavage and Linker Fragmentation.

Following oxidation, the electron-withdrawing sulfoxide groups increase the electrophilicity of the central carbon, facilitating nucleophilic attack by water and subsequent bond cleavage.

$$\left(R){}_{2}C{\left[S\left(O\right)R^{\prime }\right]}_{2}+H_{2}O\to {\left(R\right)}_{2}C=O+2R^{\prime }\mathrm{SOH}\right)$$

Where:

• $\left(R){}_{2}C=O\right)$= ketone product
• $R^{\prime }\mathrm{SOH}$= sulfenic acid intermediate

Secondary Oxidation/Dimerization of Sulfenic Acid.

Sulfenic acids are highly unstable and rapidly undergo self-condensation to form disulfides:

$$2R^{\prime }\mathrm{SOH}\to R^{\prime }\mathrm{SS}R^{\prime }+H_{2}O$$

Overall ROS-Induced Thioketal Degradation

$${\left(R\right)}_{2}C{\left(SR^{\prime }\right)}_{2}+\left[\mathrm{ROS}\right]+H_{2}O\to {\left(R\right)}_{2}C=O+R^{\prime }\mathrm{SS}R^{\prime }$$

While the mechanisms above establish the chemical feasibility of thioether and thioketal oxidation as ROS-responsive triggers, their translational relevance depends on reaction rates achievable under physiological oxidant concentrations. Thioether-to-sulfoxide oxidation by hydrogen peroxide alone proceeds with a second-order rate constant of approximately 2 × 10^−2^ M^−1^ s^−1^, corresponding to half-lives on the order of hundreds of hours at pathophysiology-relevant H₂O₂ concentrations, a timescale too slow to support rapid, on-demand release in isolation. By contrast, hypochlorite, generated by neutrophil myeloperoxidase and reaching concentrations of up to ∼400 μM in activated neutrophils, oxidizes thioethers roughly ten orders of magnitude faster (rate constant ≈ 3.7 × 10^8^ M^−1^ s^−1^), suggesting hypochlorite rather than H₂O₂ alone may be the dominant physiological trigger for thioether-based systems in inflammation-rich tumor microenvironments such as melanoma (Abdelfattah et al., 2025). Thioketal linkers show comparatively faster cleavage kinetics: metal-ion-catalyzed oxidative degradation proceeds with a half-life of approximately 2.8 h in the presence of H₂O₂, versus ∼25.3 h for the uncatalyzed reaction, while hypochlorite exposure reduces this to under 3 min. These kinetic disparities indicate that local ROS speciation, the presence of catalytic transition metals, and the specific chemistry of the responsive linker substantially influence the release timescale achievable in vivo and should be weighed alongside mechanistic feasibility when designing ROS-responsive vesicular systems for melanoma (Liu and Thayumanavan, 2020). It should be noted that these rate constants are derived from simplified chemical models under defined oxidant concentrations and buffer conditions, and their extrapolation to the complex, heterogeneous tumor microenvironment, where local pH, protein binding, and competing reactive species may all alter the effective kinetics, requires direct experimental validation.

**Fig. 4:**
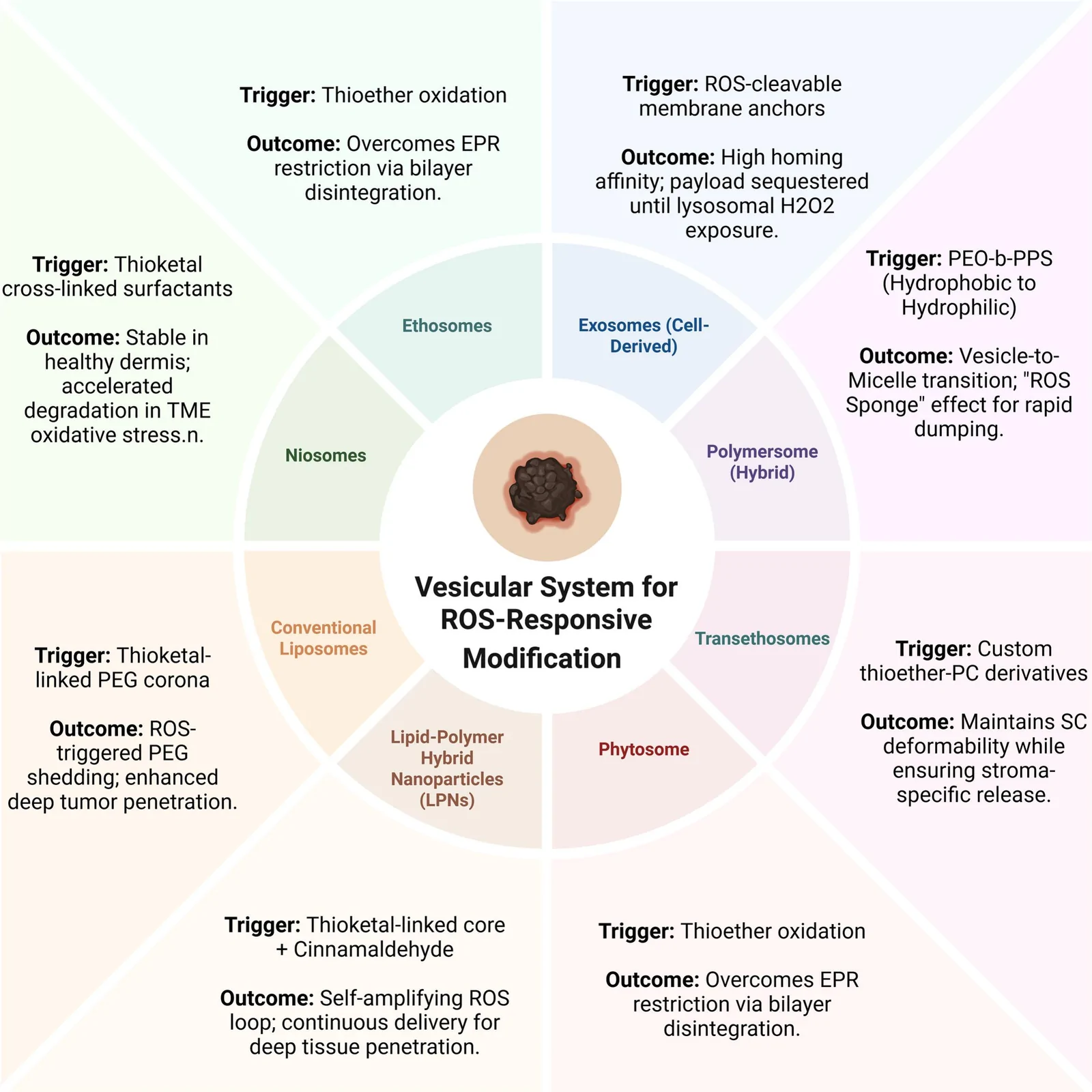
Diverse ROS-responsive vesicular architectures for targeted melanoma therapy. Overview of smart nanocarrier platforms engineered to selectively exploit the high oxidative stress inherent to the melanoma tumor microenvironment. Important structural modifications shown including thioether-mediated phase transitions, which drive a hydrophobic-to-hydrophilic shift leading to vesicle-to-micelle disassembly, and thioketal-cleavable linkers, which trigger rapid, on-demand de-PEGylation or membrane anchor shedding. These highly specific redox-sensitive mechanisms facilitate spatiotemporally regulated payload release within the malignant stroma.

Together, these ROS-responsive systems facilitate spatiotemporally regulated drug delivery and enhanced intracellular delivery. Such redox-sensitive approaches have been effectively incorporated into a broad spectrum of vesicular nanocarriers, as shown in Fig. 4, highlighting their flexibility in surmounting main biological obstacles in the treatment of melanoma.

### 3.3 Enzyme-responsive nanocarriers: MMP-triggered activation

**Fig. 5:**
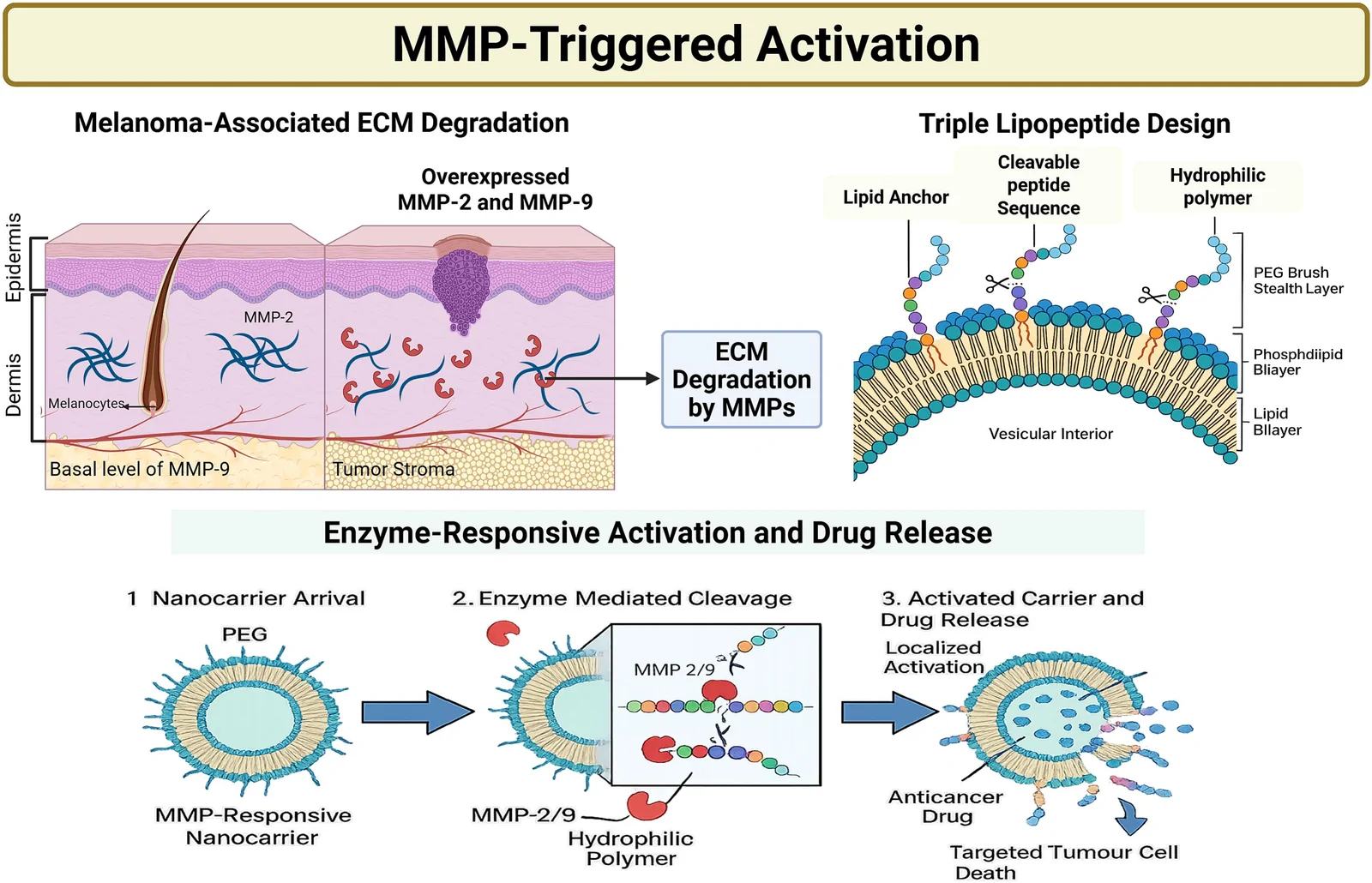
Enzyme-Responsive Activation of Nanocarriers via Matrix Metalloproteinases (MMPs). Schematic illustration of the activation of enzyme responsive vesicular carriers in the over-expressed MMP-2 and MMP-9 of the cutaneous tumor stroma. The nanocarrier is based on a triple lipopeptide structure, which includes a lipid anchor, a cleavable peptide sequence and a hydrophilic PEG polymer stealth layer. Once inside the melanoma stroma, high MMP activity immediately cleaves the peptide linkers, thereby completely removing the steric PEG corona. This unmasking event enables the release of the drug at the site and the killing of the tumor cells at the site.

Hyperactive degradation of the extracellular matrix (ECM) is a key component of the aggressive growth and metastatic spread of malignant melanoma. Consequently, matrix metalloproteinases (specifically MMP-2 and MMP-9) are overexpressed in the cutaneous tumor stroma compared to healthy skin, providing a highly specific, localized biochemical stimulus for targeted nanotherapeutics(Moni et al., 2025; Panja et al., 2025; Patra and Rengan, 2022).this mechanism has been depicted in Fig. 5. Enzyme-responsive nanocarriers exploit this overexpression by incorporating small, specific peptide sequences within the lipid membrane structure. The most commonly used technique involves the synthesis of a tripartite lipopeptide comprising a lipid anchor (e.g., DSPE), a cleavable peptide sequence (e.g., GPLGIAGQ or PLGVRG), and a hydrophilic polymer (e.g., PEG) (Chen et al., 2025; Wang et al., 2024).

#### 3.3.1 The expose-and-bind de-PEGylation mechanism

While penetrating the skin and passing through the healthy, viable epidermis (where MMP levels are low), the protective PEG coat remains intact, providing steric stabilization, preventing premature opsonization, and avoiding undesired binding to healthy melanocytes (Choi et al., 2017; Doroshenko and Shefchenko, 2025). But once the PEGylated vesicles extravasate into the melanoma TME, the high-affinity peptide linkers are swiftly recognized and digested by the elevated levels of MMPs. This cleaves the steric PEG corona completely, suddenly unmasking the underlying positive surface charges or the hidden targeting ligands (such as hyaluronic acid for CD44 receptor targeting) (Choi et al., 2017; Zhao and Li, 2025).

Step 1: MMP-2/MMP-9-Mediated Peptide Cleavage.

The peptide linker connecting the lipid and PEG moieties is specifically recognized and hydrolysed by matrix metalloproteinases (MMP-2 and MMP-9), resulting in cleavage of the conjugate.

$$\text{Lipid}-\text{Peptide}-\mathrm{PEG}+H_{2}O\overset{\mathrm{MMP}-2/\mathrm{MMP}-9}{\to }\text{Lipid}-{\text{Peptide}}_{\text{fragment}}+\mathrm{PEG}-{\text{Peptide}}_{\text{fragment}.}$$

Where:

• Lipid-Peptide-PEG = intact MMP-responsive lipid–polymer conjugate
• MMP-2/MMP-9 = matrix metalloproteinases overexpressed in the tumor microenvironment
• Lipid-Peptide _fragment_ = lipid-bound peptide fragment remaining after cleavage
• PEG-Peptide _fragment_ = PEG-bound peptide fragment released following enzymatic hydrolysis

Formulation Challenge and the Need for Statistical Rigour exist in this case, as although the therapeutic concept of MMP-cleavable vesicles is intuitively compelling, their physical formulation poses a major pharmaceutical challenge and is often overlooked in exploratory papers. The large, peptide-linked lipids attached to the vesicular membrane result in a dramatic change of the critical packing parameter (CPP) and the bilayer energy.(Fleige et al., 2012; Kulkarni et al., 2014)

The inclusion of peptide linkers risks stiffening this delicate bilayer, thereby compromising the carrier's transdermal activity before it reaches the tumor site. As such, the evolution of these smart vesicular systems, such as ethosomes, cannot proceed with the conventional, empirical “one factor at a time” (OFAT) formulation approach. To achieve an optimal balance between maximum membrane fluidity and highly specific enzymatic activation, future work must rigorously adhere to Quality by Design (QbD) principles and employ multifactorial Design of Experiments (DoE) to fine-tune the lipid-to-surfactant ratios and peptide densities(Nayak et al., 2024; Shahrukh et al., 2024).

Taken together, the single-stimulus modalities discussed above present distinct trade-offs in translational readiness and formulation complexity. pH-responsive systems are mechanistically the most mature of the three, with the largest number of Table 1 entries reporting quantitative in vivo efficacy, but their central design challenge lies in trigger reliability: extracellular tumor pH is unsuitable as a passive topical trigger due to premature activation at the acidic skin surface, and successful systems have relied instead on intracellular endosomal/lysosomal pH targeting or physical stratum corneum bypass strategies such as microneedle delivery. ROS-responsive systems are comparably well-characterized at the level of reaction chemistry, but the kinetic analysis in Section 3.2 indicates that hydrogen peroxide alone oxidizes thioether and thioketal linkers too slowly to support rapid on-demand release, making local ROS speciation, particularly hypochlorite availability in inflammation-rich regions of the tumor microenvironment, a critical, currently underexplored formulation variable. Enzyme-responsive systems offer the most spatially specific trigger of the three, since MMP-2/9 overexpression is tightly localized to the tumor-stroma interface, but they are the least represented among Table 1 with complete efficacy data and carry a distinct formulation risk: the peptide linkers required for enzymatic cleavage can stiffen the vesicle bilayer, compromising the deformability needed for transdermal permeation before the carrier ever reaches its intended enzymatic trigger. No single stimulus modality is therefore unambiguously superior across all three axes of reliability, translational readiness, and formulation compatibility, a limitation that motivates the multi-stimuli combinatorial strategies discussed in further sections.

**Table 1:** In vivo and in vitro preclinical efficacy of single-stimulus responsive vesicular systems for melanoma therapy.

Type of Vesicular Carrier	Stimuli-Responsive Mechanism	Quantitative Melanoma Growth Reduction (In Vivo)	Quantitative IC_50_ Values (In Vitro)	Reference
Liposomes (TR and C18-TR modified) (IV) ^+^	pH-sensitive (charge conversion at acidic tumor microenvironment pH 6.3)	Tumor reduction ratios of 39.4% (PTX-LIP^TR^) and 56.1% (PTX-LIP^C18-TR^) compared to 20.8% for unmodified liposomes.	12.85 μM (PTX-LIP^TR^) and 10.80 μM (PTX-LIP^C18-TR^) in B16F10 cells at pH 6.3.	(Huang et al., 2023)
Liposomes (CTX@PSL) (IP)^+^	pH-responsive (protonation of CHEMS in acidic tumor environment pH 6.5–6.8)	Tumor weight was reduced to 0.47 ± 0.12 g (compared to 2.32 ± 0.89 g for control), resulting in a tumor inhibition rate of 79.78 ± 5.93%.	0.25 μg/mL in B16-F10 cells.	(Lin et al., 2024)
Liposomes (cRGD-LP-HCPT) within microneedle patches ^+^	pH-sensitive (CHEMS protonation at acidic pH)	Median survival time extended to 18 days (vs. 12 days for free drug); survival rate reached 40% on day 24.	1 μg/mL in B16F10 cells.	(Zhou et al., 2025)
Histidinylated cationic liposomes*	Endosomal pH-sensitive (protonation of imidazole head-groups at pH 5.5–6.5)	Suppressed melanoma volume growth to ∼2000 mm^3^ compared to ∼8000 mm^3^ in the untreated control group at day 27.	Not quantitatively reported (demonstrated significant late apoptosis).	(Garu et al., 2015)
Multifunctional vesicles (LA-BPA) (IV)^+^	pH-dependent reversible assembly & Neutron irradiation (BNCT)	Extended overall survival time to >35 days compared to 18 days for the untreated control group.	Not reported for melanoma (only reported for pancreatic/breast lines).	(Dai et al., 2025)
Sterically stabilised cationic liposomes (SSCL)*	pH-sensitive (destabilization at acidic endosomal pH)	Suppressed tumor volume to ∼450 mm^3^ (regression) at day 18, compared to ∼1500 mm^3^ for the naive group (reduced tumor size by 70%)	Not quantitatively reported (assessed via immune activation and cytokine production)	(Kocabas et al., 2020)
Liposome-encapsulated ruthenium (LipoRu) in transdermal hydrogel	Near-infrared (NIR) light-triggered (Two-photon excitation)	Inhibited tumor growth by 61% (intratumoral), 78% (intravenous), and 67% (transdermal)	0.143 ± 0.015 μM (Ru2 complex) and 0.086 ± 0.011 μM (Ru3 complex) under two-photon excitation IC_50_ for LipoRu under two-photon excitation is 0.116 ± 0.010 μM	(Yan et al., 2025)

Note: For entries marked with an asterisk (*), in vitro efficacy was assessed via qualitative endpoints (apoptosis induction, immune/cytokine activation) rather than a quantitative IC₅₀ value, as reported in the original source.

Entries marked + achieved efficacy via systemic, intratumoral, or microneedle-assisted administration, circumventing rather than resolving the topical bio-relevance gap discussed in Section 3.1.3.

## 4 Multi-stimuli responsive and combinatorial nanocarriers

Although single-stimulus-responsive nanocarriers represent a major improvement over passive delivery systems, their clinical efficacy is often hampered by the strong spatiotemporal heterogeneity of the melanoma tumor microenvironment(Chen et al., 2022; Zhang et al., 2025). The premature leakage of the payload into off-target tissues undergoing transient inflammation, or vice versa, can result from a dependence on a single pathophysiological trigger, such as mild extracellular acidosis or elevated baseline oxidative stress(Pandey et al., 2026). To address this intrinsic shortcoming, modern formulation approaches have gradually shifted toward multi-stimuli-responsive and combinatorial nanocarriers. These highly advanced vesicular structures are designed to sense and respond to multiple biological signals either in a cascade or in a highly localized therapeutic index.

The mechanistic basis of multi-stimuli systems relies on the application of biological Boolean logic, most notably the “AND” gate principle(Bressler et al., 2023). In this design, the nanocarrier must receive at least two distinct stimuli to undergo complete structural disassembly and drug release(Badeau et al., 2018). A primary paradigm is the sequential dual-responsive carrier (e.g., enzyme/pH). These systems frequently feature a protective, stealth polyethene glycol (PEG) corona tethered to the vesicular bilayer by a matrix metalloproteinase (MMP)-cleavable peptide linker, encasing a core functionalized with protonatable, charge-reversing lipids. Upon entering the melanoma stroma, localized MMP overexpression initiates the first trigger, shedding the PEG corona to unveil underlying targeting ligands. Following this critical unmasking and subsequent cellular endocytosis, the precipitous decrease in endolysosomal pH serves as the second trigger, orchestrating charge reversal, endosomal escape, and the highly localized release of the chemotherapeutic payload into the cytosol(Liang et al., 2024).

However, simultaneous dual-intracellular triggers, such as pH/ROS systems, take advantage of the simultaneous appearance of severe endosomal acidosis and increased oxidative stress in the cytoplasm (Chang et al., 2020). These carriers can be modified to undergo a faster transition to the internalized state and thus evade multidrug resistance caused by drug efflux pumps (Li et al., 2021). Moreover, triple-responsive systems have been developed that integrate endogenous TME cues with exogenous stimuli, including near-infrared (NIR) irradiation, to enable targeted photothermal therapy along with simultaneous (on-demand) chemotherapy (Ou et al., 2025; Zhang et al., 2024b).

However, there are profound biophysical constraints on multi-stimuli carriers even if the theoretical advantages are indisputable. Multiple different functional groups get conjugated to each other, the synthetic complexity is raised to an exponential power, and the critical packing parameters of the lipid bilayer are disturbed. These beautifully decorated vesicles often suffer from poor baseline stability, weak deformability during passage through the stratum corneum and poor encapsulation efficiency, thus highlighting the need to balance conceptual elegance with transdermal viability. This convergence of active-targeting ligand functionalization with stimuli-responsive release has been highlighted as a key direction for liposomal melanoma therapeutics (de Paulo et al., 2026). This tension between synthetic sophistication and physical deliverability constitutes a broader formulation paradox for multi-stimuli carriers, echoing the same optimisation challenge identified for MMP-cleavable systems in Section 3.3. Rather than pursuing complexity empirically, future multi-stimuli designs should adopt Quality by Design (QbD) frameworks combined with multifactorial Design of Experiments (DoE) to systematically map the interdependencies between conjugation density, lipid packing, and transdermal deformability, allowing formulators to identify an optimal balance point rather than defaulting to maximal stimulus-responsiveness at the expense of deliverability.

## 5 Clinical translation and future perspectives

Although there has been an exponential increase in preclinical literature on the in vitro and in vivo efficacy of stimuli-responsive transdermal nanocarriers, this has not yet translated into clinically approved oncological therapies, as shown in Table 2. This translational drawback is driven by a convergence of severe manufacturing bottlenecks, regulatory complexities, and fundamental flaws in traditional preclinical validation paradigms. A primary obstacle to commercialisation is the technical complexity of achieving large-scale, Good Manufacturing Practice (GMP)-compliant production. Multi-step, custom bioconjugation chemistries are typically used to synthesise precisely engineered stimuli-cleavable lipids or lipopeptides. The scaling of these processes between microfluidic bench-top continuous-flow systems or thin-film hydration and industrial volumes often leads to unacceptable batch-to-batch variability in vesicle size, lamellarity, and stimulus responsiveness.

**Table 2:** Summary of clinical trials investigating vesicular nanotechnology systems in melanoma treatment.

NCT Number	Vesicular System	Phase	Stimuli-Responsive	Key Findings	Primary Translational Limitation	Primary Failure Mode
**NCT00089180**^a^	T4N5 liposomal lotion (topical, pH-sensitive vehicle)	Phase II/III	Passive only	Actinic keratosis rate reduced by 68%; basal cell carcinoma incidence reduced by 30% vs placebo over 1 year in xeroderma pigmentosum patients	Restricted to DNA repair enzyme delivery; no cytotoxic payload; demonstrates topical liposome feasibility but not anti-melanoma therapeutic efficacy	N/A (non-melanoma indication; not an efficacy trial)
**NCT01052142**	Lipovaxin-MM (DC-targeted liposomal vaccine)	Phase I	No	Well tolerated; no DLT; no vaccine-specific immune response detected; 1 partial response, 2 stable disease in 12 patients	Passive liposomal vehicle unable to overcome endosomal degradation; insufficient intracellular antigen delivery; failure to elicit immunogenicity	Poor efficacy (failed to elicit immunogenicity)
**NCT00145041**	Liposomal Vincristine (VSLI)	Phase I/II	No	Pharmacokinetic study in 12 melanoma patients with hepatic impairment; PK parameters similar in patients with impaired vs. adequate liver function; VSLI generally well tolerated	Systemic administration; no tumor selectivity; neurotoxicity ceiling limits dose escalation; passive EPR-dependent accumulation	N/A (pharmacokinetic/safety study only)
**NCT00506142**	Liposomal Vincristine (Marqibo)	Phase II	No	Phase II study in metastatic uveal melanoma; PK and safety assessment; efficacy results not publicly reported in primary literature	Heterogeneous EPR-dependent tumor uptake in desmoplastic stroma; insufficient intratumoral drug concentration at tolerable doses^b^	Poor efficacy (inconclusive; efficacy not established)
**NCT02862145**	MRX34 (miR-34a liposomal injection)	Phase I	No	Withdrawn; severe immune-mediated adverse events in 4 patients, including 1 death	Cationic lipid vehicle triggered TLR3-associated innate immune activation (dsRNA/miRNA-mimic sensing), resulting in severe immune-mediated adverse events including fatality; toxicity was immunological in origin rather than attributable to absence of charge-switching.	Toxicity (immune-mediated)
**NCT01829971**	MRX34 (miR-RX34 liposomal injection)	Phase I	No	Terminated; recurrent immune-mediated adverse events	Same platform as NCT02862145; confirms toxicity is immune-pathway-related rather than patient-specific; underscores the need for immunologically inert, non-cationic delivery systems rather than charge-switching alone.	Toxicity (immune-mediated)
**NCT01563614**	Liposomal cytarabine	Phase I/II	No	Terminated; insufficient anti-melanoma activity	Drug insufficiently active in melanoma biology; passive delivery failed to achieve cytotoxic intracellular threshold without TME-exploiting mechanism	Poor efficacy
**NCT04244552**	Pegylated liposomal doxorubicin (PLD)	Phase II	No	Terminated; insufficient efficacy in melanoma	Permanent PEG coating without cleavable de-PEGylation impairs cellular uptake; passive EPR unreliable in high-IFP desmoplastic melanoma stroma	Poor efficacy (formulation-related)
**NCT05264974**	DOTAP liposomal mRNA vaccine (RNA-LP)	Phase I	No	Suspended	mRNA instability during processing and storage; manufacturing complexity; absence of tumor-selective activation mechanism	Manufacturing (CMC/stability)

a Included as an early precedent for topical liposomal vehicle feasibility in cutaneous oncology chemoprevention (xeroderma pigmentosum/actinic keratosis), not as a melanoma-treatment efficacy trial.

b Marqibo/VSLI is FDA-approved for Philadelphia chromosome-negative acute lymphoblastic leukemia; this entry refers specifically to its investigational, off-label Phase II evaluation in metastatic uveal melanoma, for which efficacy was not established.

Moreover, such multi-component, complex excipients are always non-compendial. Regulatory-wise, global health regulators like the FDA and EMA demand exhaustive Chemistry, Manufacturing, and Controls (CMC) documentation, and rigorous toxicological profiling of all new synthetic constituents. A clever nanocarrier designed to be fundamentally unstable under certain physiological conditions ironically faces insurmountable challenges in demonstrating the necessary long-term shelf stability, as functional groups such as thioethers or hydrazone bonds are highly prone to premature degradation during extended storage. This regulatory burden is thus compounded, and a potentially promising therapeutic becomes a commercially unfeasible venture due to prohibitive developmental costs and lengthy approval processes.

No less harmful to clinical translation is the widespread dependence on poor preclinical models. Two-dimensional cell cultures and subcutaneous murine xenografts are not representative of the two biophysical barriers that transdermal nanocarriers need to overcome in humans. Rodents have a very thin stratum corneum, a higher density of appendages, and naturally different lipid profiles, all of which yields an enormous overestimation of the transdermal permeation efficacy. Furthermore, subcutaneous xenografts do not accurately reflect the dense, desmoplastic architecture, high interstitial fluid pressure, and unique immune context of spontaneous human cutaneous melanoma. Future studies will require more aggressive translation away from murine models to humanized three-dimensional skin equivalents, ex vivo human skin explants and advanced microfluidic platforms to overcome this translational disadvantage. In particular, it is important that perfusable, vascularized melanoma-on-a-chip systems are integrated, since they can accurately mimic the forces of the human TME, such as interstitial fluid pressure and complex shear stress, which regulate nanocarrier extravasation, but are completely missing in static cultures.Furthermore, the adoption of multi-organ microfluidic models will be essential to evaluate early metastatic dissemination cascades and off-target nanocarrier toxicities with a degree of human bio-relevance that standard xenografts fundamentally cannot provide.

Beyond the stimuli-responsive strategies discussed here, complementary directions warrant consideration: nanocarrier-mediated modulation of autophagic pathways represents an emerging adjunct mechanism for enhancing intracellular drug action (Sun et al., 2025); combination with immunotherapeutic approaches that address tumor-mediated immune evasion, such as NK-cell-based strategies, may further improve therapeutic outcomes when paired with targeted nanocarrier delivery (Zheng et al., 2026); and non-invasive imaging modalities such as PET/CT, already used to monitor response in cellular immunotherapies, may eventually support real-time assessment of nanocarrier-based combination regimens (Singh et al., 2024)

## 6 Conclusion

The therapeutic challenge of cutaneous melanoma is even more challenging, not only because of its biological aggressiveness, but because of its dual biophysical architecture, which must be overcome by any topical therapy. The interface between the stratum corneum and the melanoma tumor microenvironment isn't a research gap that needs to be overcome step-by-step, but rather a single design constraint that has been a major barrier to the clinical translation of traditional passive nanocarriers. This review suggests that stimuli-responsive vesicular systems represent the first rational pharmaco-technological approach to overcome both issues: robust deformability and stealthiness during SC transit, and precise, programmed structural disassembly upon encountering the aberrant pathophysiological hallmarks of the malignant stroma.

Each of the three mechanistic classes explored exploits a unique and localized biochemical signature of the melanoma TME, namely pH-responsive charge reversal and acid-cleavable de-PEGylation, ROS-triggered thioether and thioketal phase transitions and MMP-mediated lipopeptide cleavage. It was found, however, that there is a bio-relevance paradox inherent in the transdermal route that has not been widely recognized in the literature: systems designed to respond to extracellular tumor acidosis (pH 6.2–6.8) are inherently susceptible to premature disassembly at the highly acidic skin surface (pH 4.5–5.5), making extracellular pH an unreliable trigger for topical oncology. A change in design that would have major implications for the choice of protonatable head-group and pKa engineering, as well as implications for lipid composition, is the need for future pH responsive transdermal designs to activate in the intracellular lysosomal compartment (pH <5.0).

The multi-stimuli responsive systems described are the next step in this paradigm, combining biological AND-gates to increase selectivity and minimize off-target activation. However, adding synthetic complexity, as in multi-functional surface modification, comes with its own formulation paradox: the stimulus specificity of the surface can potentially disrupt membrane fluidity, hindering effective SC penetration. One of the great unmet challenges in this area is to reconcile responsiveness with deformability, thus achieving rigorous Quality by Design approaches and multifactorial optimization.

Eventually, three systemic issues that pre-clinical literature has long postponed will need to be addressed through deep research to enable progress in translation. First, the synthesis of stimuli-cleavable lipids/lipopeptides at industrial volumes remains unresolved due to high batch-to-batch variability in the bench-top microfluidic process and thin-film thickness uniformity. Second, non-compendial and intrinsically unstable excipients require optimization of both stability on the shelf as well as stimulus responsiveness, which are chemically contradictory and will demand the development of novel excipient stabilization methods, including the use of lyophilization with cryoprotectants or encapsulation in secondary protective matrices. Third, and most important, the field should move beyond the use of subcutaneous murine xenografts to humanized 3D skin equivalents, ex vivo human skin explants and vascularized melanoma-on-a-chip platforms which can recapitulate the dual barrier physiology of the human clinical target.

The stimulus-responsive vesicular nanocarrier is a promising combination of pharmaceutical nanotechnology, cancer biology, and precision medicine. To realize its clinical potential, it will require the field to go beyond proof-of-concept demonstrations and address more challenges, including scalable manufacturing, mechanistically sound preclinical validation and pathway engineering for regulatory approval. The conceptual paradigm outlined here, in which both the deformability of the SC and its activation by the TME are treated as equal, interdependent design goals, lays the groundwork for the next step in the translation.

## Declaration of generative AI and AI-assisted technologies in the manuscript preparation process

During the preparation of this work, the author(s) used Grammarly, and QuillBot to support language refinement, grammar correction, and improvement of sentence clarity and readability. After using these tools, the author(s) reviewed, edited, and validated all content manually to ensure accuracy, originality, and integrity of the final manuscript. The author(s) take full responsibility for the content of the published article.

## CRediT authorship contribution statement

**Rudresh Adarkar:** Writing – review & editing, Writing – original draft, Visualization, Data curation, Conceptualization. **Akanksha Dessai:** Writing – review & editing. **Manoj Madanahalli Ramesh:** Writing – review & editing. **Richard Lobo:** Supervision. **Vamshi Krishna Tippavajhala:** Supervision. **Chandrashekar Kodangala Subraya:** Supervision, Investigation, Conceptualization.

## Funding

This research received no external funding.

## Declaration of competing interest

The authors declare that they have no known competing financial interests or personal relationships that could have appeared to influence the work reported in this paper.

## Data Availability

No new primary data were generated or analyzed in this study. This article is a review of previously published literature, and all data discussed are available in the cited primary sources.
